# Co-occurrence of High-Risk Myelodysplastic Syndrome With a Complex Karyotype/TP53 Mutation and IgG Lambda Monoclonal Gammopathy of Undetermined Significance

**DOI:** 10.7759/cureus.58451

**Published:** 2024-04-17

**Authors:** Marwa Mir, Wajeeha Aiman, Esther Baah, Gunwant Guron, Hamid Shaaban

**Affiliations:** 1 Internal Medicine, Saint Michael's Medical Center, Newark, USA; 2 Internal Medicine, St. George's University School of Medicine, St. George's, GRD; 3 Hematology and Oncology, Saint Michael's Medical Center, Newark, USA; 4 Hematology and Oncology, New York Medical College, Valhalla, USA

**Keywords:** igg lambda, complex karyotype, tp53 mutation, monoclonal gammopathy of unknown significance, myelodysplastic syndrome

## Abstract

Myelodysplastic syndrome (MDS) represents a group of hematologic disorders marked by abnormal cellular development in the bone marrow, while monoclonal gammopathy of undetermined significance (MGUS) involves abnormal plasma cells without symptomatic manifestations. This paper presents a compelling case of a 74-year-old Hispanic female diagnosed with a rare combination of high-risk MDS characterized by a complex karyotype and TP53 mutation, alongside IgG lambda MGUS. The patient's clinical presentation included a spectrum of symptoms such as body aches, rash, fever, dyspnea, and bloody watery diarrhea. Initial diagnostic evaluations yielded no significant findings, but subsequent investigations revealed abnormalities in both bone marrow and peripheral blood, indicative of coexisting MDS and MGUS. Chromosomal analysis further confirmed the presence of a complex karyotype with multiple aberrations, notably including 5q deletion. This case underscores the rarity of simultaneous high-risk MDS and MGUS, particularly with the additional complexity of a TP53 mutation and complex karyotype. It underscores the imperative for continued research efforts to elucidate the underlying mechanisms and optimal management strategies for such intricate cases. Moreover, it highlights the therapeutic challenges posed by concurrent MDS and plasma cell disorders, advocating for more aggressive interventions such as stem cell transplantation as potential avenues for improved patient outcomes.

## Introduction

Myelodysplastic syndromes (MDS) are a set of hematologic malignancies associated with persistent cytopenia and characterized by a failure of proper hematopoietic cellular maturation. MDS can be associated with an increased risk of progression to acute myeloid leukemia (AML) and other malignancies. The incidence of MDS is approximately four in every 100,000 people. The condition is diagnosed when there is unexplained chronic cytopenia, less than 20% blasts in peripheral blood/bone marrow, and characteristic cytogenetic or dysplastic morphology [1,2]. MDS are often associated with 5q and 7q deletions, trisomy 8, and several monosomies [2]. MDS can be associated with monoclonal gammopathy of undetermined significance (MGUS), a non-neoplastic plasma cell disorder that presents with symptomless clinical manifestations. MGUS is defined by a serum monoclonal protein (M protein) of 3 g/dL, a bone marrow plasmacytosis of less than 10% monoclonal plasma cells, and a lack of end-organ damage [3,4]. MGUS has three distinct subtypes: non-IgM MGUS (IgG, IgA, or IgD MGUS), IgM MGUS, and light-chain MGUS [5,6]. Non-IgM is determined by a serum M protein 3g/dL; 10% clonal plasma cells in the bone marrow; and the absence of hypercalcemia, renal dysfunction, anemia, and bone lytic lesions (CRAB) [6]. IgM MGUS has the same criteria as non-IgM MGUS plus the absence of constitutional symptoms, hyperviscosity, lymphadenopathy, and hepatosplenomegaly. Light-chain MGUS is characterized by an abnormal free light-chain (FLC) ratio, increased light chains (high kappa light chain when the FLC ratio is high or high lambda light chains when the FLC ratio is low), a lack of monoclonal IG heavy chains, and an absence of CRAB. MGUS affects about 1%-2% of the US adult population [7]. The average age of diagnosis is 70 years old, and the incidence increases with age. The prevalence of MGUS also increases with age, with 3.3% of those ≥50 years affected, 5.3% of those ≥70 years, and 7.5% of those ≥85 years of age. Prevalence is also influenced by sex, with 4% in women over 50 and 2.7% in men over 50 years of age [8-10]. MGUS can sometimes progress to MDS, although there is limited data on the concurrence of both syndromes. It is a unique but well-known entity with noted molecular and phenotypical characteristics. There is a case report that discussed a patient with MGUS that progressed to MDS with 5q deletion and severe pancytopenia [9]. The occurrence of MGUS progressing to MDS is rare when compared to its progression to multiple myeloma (MM) [11]. Concomitant MDS with MGUS occurs in about 10%-15% of MDS cases [11,12]. A study by Kewan et al. depicted that, out of 1627 MDS patients, 6% had concurrent MGUS, and those with both diseases had a median age of 76 years of age. The dyad of MDS/MGUS may purport a better outcome and overall increased survival rate when compared to MDS without MGUS [12]. Here, we present a rare case of a patient with extremely high-risk MDS with complex karyotype and bone marrow blasts between 5% and 10%, pancytopenia, and concomitant MGUS.

## Case presentation

We present a case of a 74-year-old Hispanic female with a past medical history of type 2 diabetes mellitus, dyslipidemia, osteoporosis, and hypothyroidism first presented with complaints of diffuse body aches, right postauricular sharp pain that radiated to her neck and jaw, fever of 102 degrees Fahrenheit, dyspnea, maculopapular rash, and bloody diarrhea. She is originally from Ecuador and immigrated to the United States in 2003 but has a recent history of travel and a six-week stay in Ecuador prior to presentation. On admission, she was hemodynamically stable but leukopenic and thus started on empiric antibiotics. In addition, the patient had a macrocytic anemic with hemoglobin of 7.1 and mean corpuscular volume (MCV) of 102, requiring one unit of packed red blood cells to be transfused. The iron profile and b12 and folate levels were all within normal limits. She became febrile for three consecutive nights, which prompted a deeper probing with an infectious and autoimmune workup.

Initial tests and imaging including HIV, fecal occult blood test, computed tomography (CT) angiogram of the abdomen and pelvis, and abdominal ultrasound proved to be unremarkable. Her CT chest was positive for pleural effusions. Her urine culture was positive, but she remained asymptomatic. The autoimmune panel yielded negative antinuclear antibody (ANA) and rheumatoid factor (RF). Peripheral blood smear showed atypical white blood cells, leukocytopenia, neutropenia, and anemia with macrocytic and hyperchromic red blood cells.

She was then discharged after clinical improvement in her symptoms and instructed to follow up for pancytopenia workup as an outpatient. However, she returned a few days later with complaints of persistent fever, generalized fatigue, myalgia, decreased appetite, intermittent non-bloody diarrhea, and exertional dyspnea associated with orthostatic syncope.

**Figure 1:**
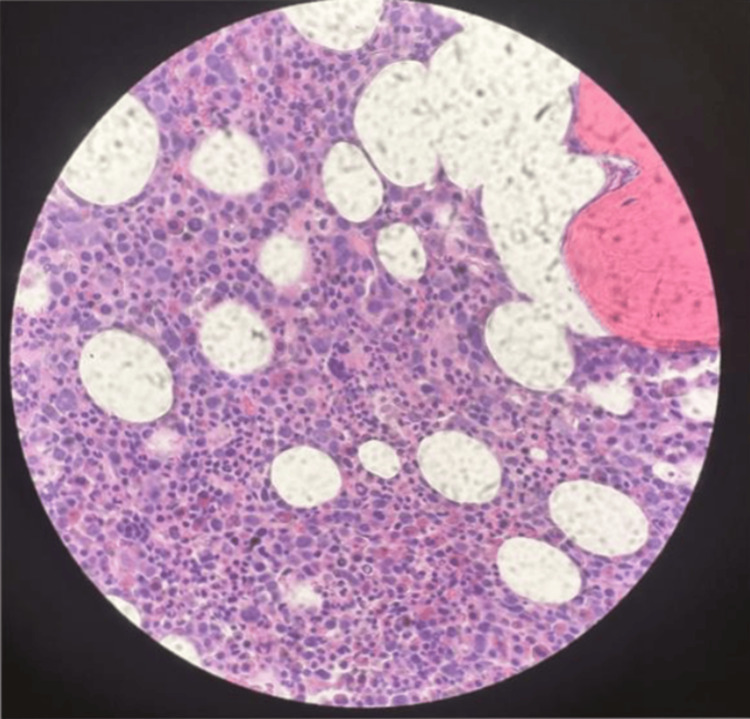
Bone marrow core biopsy demonstrates a hypercellular marrow for age (70%) with left-shifted myeloid hematopoiesis and an increased myeloid and erythroid lineage ratio (M:E) due to myeloid overgrowth, multilineage myelodysplastic syndrome, and increased myeloblasts (5%-10%) (high power field).

During this admission, a bone marrow core biopsy, bone marrow aspirate, peripheral blood smear, and chromosomal analysis were conducted. The biopsy and aspirate results (Figure 1) revealed hypercellular marrow for age (70%) with left-shifted myeloid hematopoiesis and an increased myeloid (M) and erythroid (E) lineage ratio (M:E) due to myeloid overgrowth. There was an increased number of megakaryocytes, and they also exhibited cytologic dysplasia. Prussian blue stain showed focal stainable iron without sideroblasts. Reticulin stains showed no significant increase in reticulin fibers. Immunohistochemical stains showed scattered CD138-positive plasma cells (5%-10% of total cells). Scattered B-cells and T-cell lymphocytes were highlighted by CD20 and CD3, respectively. Blasts were noted by CD34 immunostaining (5%-10% of total cells). MPO, CD71, and CD81 stains highlighted myeloid cells, erythroid cells, and megakaryocytes, respectively. The CD117 stain highlighted a good number of positive cells, and the p53 stain labeled many positive cells (30%). She also had blood clots with a small focus of minute bone marrow spicules exhibiting hypercellular marrow with left-shifted myeloid overgrowth. Her peripheral blood and flow cytometry revealed a mild increase in blasts (3.5%) and monocytosis (9%). We identified scattered polytypic plasma cells (5%-10%) in core biopsy and clots.

In the sample analyzed, there was no detectable clonal plasma cell population. Fluorescence in-situ hybridization (FISH) studies for MDS and MM panels were positive for trisomy 7. FISH MDS panel depicted a deletion of CSF1R/RPS14 on the long arm of chromosome 5 and a deletion of MDFIC on the long arm of chromosome 7.

With the presence of IgG lambda gammopathy by immunofixation electrophoresis and positivity of trisomy 7 in enriched plasma cells, plasma cell dyscrasia cannot be completely excluded. The patient was followed up in the oncology office and was started on decitabine-cedazuridine (DC) 35-100 mg for high-grade MDS with a very complex karyotype and TP53 mutation.

The patient was scheduled for a forthcoming bone marrow transplant. Additionally, she periodically required blood transfusions along with treatment.

## Discussion

Our case is unique in that it is, to our knowledge, the first case of high-risk MDS with a complex karyotype/TP53 mutation association with an IgG lambda monoclonal gammopathy of unknown significance.

The cytogenetic features associated with MDS are crucial for determining diagnosis, prognosis, and therapy. Cytogenic aberrations are a common occurrence in MDS and are present in about 50% of MDS cases. The most common of these aberrations are 5q deletions, abnormalities in chromosome 7, trisomy 8, 20q deletions, and the absence of the Y chromosome [13-17]. To the best of our knowledge, there is a paucity of data regarding high-risk MDS with a complex karyotype and concurrent MGUS with a high risk of progression to AML.

Yan et al.'s study explored the uncommon co-occurrence of myeloid neoplasia, specifically myelodysplastic syndrome with excess blasts (MDS-EB), and plasma cell neoplasia (such as MM or MGUS) with a prevalence of 0.31-1.1% [17]. After examining 34 published instances and analyzing seven case reports, the study indicated that patients with these disorders frequently develop AML after suffering from severe anemia or pancytopenia. Significantly, two separate variations malignant myeloid and plasma were discovered, together with osteolytic lesions. Unfortunately, many of the therapy responses indicate either no response at all or a swift recurrence. Considering there are no proven treatments for these individuals, the median overall survival is barely eight months, underscoring the critical need for more research and focused therapeutic approaches [14].

A challenging clinical scenario arises when MDS coexists with plasma cell malignancies, such as MM or MGUS. AML and cytopenia are the main outcomes of MDS, whereas organ failure and monoclonal gammopathy are prevalent in plasma cell disorders. The chance of developing MDS or other secondary malignancies may increase with MM treatment. In certain cases, aberrant myeloid cells and plasma cells are present at the time of diagnosis, suggesting genetic origins. Comprehending this atypical dual neoplasm is essential for efficacious therapy strategies [15].

Chemoresistance in patients with MGUS/MM and concurrent MDS-EB led to the recommendation of more aggressive therapies, including the combination of immunomodulators, monoclonal antibodies, and proteasome inhibitors. It is suggested that early allogeneic stem cell transplantation will increase overall survival.

There is some existing data suggesting that the concurrency of MDS and MGUS may have improved survival. The study by Kewan et al. [12] found that MDS/MGUS may have increased the frequency of CEBPA and STAT3 mutations, but not in other common mutations. The same study showed a 21% improved prognosis in the MDS/MGUS arm, as opposed to those without MGUS. They also found that concurrent MDS/MGUS had a 5% increase in the occurrence of secondary AML [12,16]. There seems to be a notable association between the concurrence of both diseases, and further studies into the concurrence may provide invaluable guidance on cytogenic aberrations and prognosis.

Tumor-suppressor protein p53 is essential for maintaining the genetic integrity of cells and controlling important functions such as cell division, DNA repair, and apoptosis. Within the field of plasma cell dyscrasia, which includes diseases such as MM and Waldenstrom's macroglobulinemia, the significance of TP53 mutations is especially evident. Its critical function is particularly evident in preventing the conversion of healthy plasma cells into cancerous ones. In plasma cell dyscrasia, TP53 mutations and deletions are correlated with genomic abnormalities. These changes impair cell cycle regulation, which aids in the emergence and advancement of illnesses involving plasma cells [17,18].

In lymphoid illnesses, including those involving plasma cells, TP53 mutations, especially those in the DNA-binding domain, increase the risk of aggressive disease, worse treatment outcomes, and shorter survival spans. The mutations in TP53 that occur in plasma cell dyscrasia have an immense effect on prognosis, therapy choices, and overall survival. Despite the difficulties caused by TP53 mutations, current studies investigate potential treatments to address p53 system abnormalities. Treatment alternatives CP31398, ibrutinib, Nutlin-3a, and Prima Met are being investigated for their potential to circumvent TP53 mutations [19]. Comprehending the importance of TP53 is essential for customizing targeted treatments and improving overall patient care and results. IL‐6 is a potent human myeloma‐cell growth factor, and its overproduction is known to play a critical role as an anti‐apoptosis‐inducing agent in multiple myeloma. It is possible that there is an increased number of strongly p53‐positive plasma cells in patients with concomitant MGUS and MDS, causing an increase in serum IL‐6 levels, which may be of clinical significance when it comes to treatment options and prognosis [20].

There is a genetic connection between MDS and AML resulting from MDS, and the presence of certain genetic mutations displays a continuum of disease progression. The frequency of certain mutations provides evidence of clonal evolution, and sequencing of such expansion can aid in the detection of disease advancement before it is clinically evident [14]. Certain clonal expansions during transformation may be evident once the progression to AML has occurred but not once there is sole MDS. There seems to be a role for further investigation into stem cell compartments in the progression of MDS to AML and the approach to treatment [15]. In addition, the presence of a complex karyotype (as seen in our patient) is a poor prognostic factor and is a common finding in the progression of MDS to AML. It was found that about 30% of spontaneous MDS is accompanied by a complex karyotype [16]. The presence of a complex karyotype necessitates the classification of high-risk MDS on the International Prognostic Scoring System (IPSS) classification system. The gravity of complex karyotypes in MDS and its presence in case patients further supports the need for additional information in adding to the data on the treatment and prognosis of these patients.

## Conclusions

This is a rare case of a simultaneous diagnosis of high-risk MDS with a complex karyotype and a TP53 mutation associated with an IgG lambda monoclonal gammopathy. Patients with MDS undergo a thorough screening for plasma cell dyscrasias, particularly if the existence of such disorders may have an influence on the prognosis. The coexistence of these two hematological malignancies is clinically uncommon with a paucity of case reports, unknown mechanisms, unclear treatment approaches, and an uncertain prognosis. This case report contributes to medical literature. This rare disease entity with distinct clinical and molecular features has a poor prognosis, and more studies are needed to understand how to treat it.
